# ANXA1 as a Prognostic and Immune Microenvironmental Marker for Gliomas Based on Transcriptomic Analysis and Experimental Validation

**DOI:** 10.3389/fcell.2021.659080

**Published:** 2021-08-04

**Authors:** Zhongxiao Lin, Min Wen, Enxing Yu, Xiao Lin, Hua Wang, Jiayu Chen, ChaoJie Yao, Hengli Zhang, Junnan Ru, Kankai Wang, Ying Zhang, Lijie Huang, Qichuan Zhuge, Su Yang

**Affiliations:** ^1^Zhejiang Provincial Key Laboratory of Aging and Neurological Disorder Research, Department of Neurosurgery, The First Affiliated Hospital of Wenzhou Medical University, Wenzhou, China; ^2^Department of Neurosurgery, School of Medicine, Guangzhou First People’s Hospital, South China University of Technology, Guangzhou, China

**Keywords:** glioma, tumor microenvironment, annexin A1, prognostic signature, prognostic indicator

## Abstract

The tumor microenvironment (TME) plays an important role in the growth and invasion of glioma. This study aimed to analyze the composition of the immune microenvironment in glioma samples and analyze the important differentially expressed genes to identify novel immune-targeted therapy for glioma. We downloaded transcriptomic data of 669 glioma samples from The Cancer Genome Atlas database. CIBERSORT and ESTIMATE methods were used to calculate the proportion of tumor-infiltrating immune cells and ratio of immune and stromal components in the TME. The differentially expressed genes (DEGs) were screened by comparing the genes expressed by both stromal and immune cells. Annexin A1 (ANXA1) was determined to be an important prognostic indicator through the common overlap of univariate Cox regression analysis and protein–protein interaction network analysis. The proportion of tumor-infiltrating immune cells, calculated by CIBERSORT algorithm, had a significant difference in distribution among the high and low ANXA1 expression groups, indicating that ANXA1 could be an important immune marker of TME. Furthermore, ANXA1 level was positively correlated with the histopathological factors and negatively related to the survival of glioma patients based on the analysis of multiple databases. Finally, *in vitro* experiments verified that antagonizing ANXA1 expression promoted cell apoptosis and inhibited the invasion and migration capacities of glioma cells. Therefore, ANXA1 due to its immune-related functions, can be an important prognostic indicator and immune microenvironmental marker for gliomas. Further studies are warranted to confirm ANXA1 as a potential immunotherapeutic target for gliomas.

## Introduction

Glioma is the most common primary intracranial tumor, characterized by high recurrence, easy invasion, and high mortality (Ostrom et al., 2014). Currently, the treatment for gliomas includes surgical resection, chemotherapy, and radiotherapy; however, the prognosis remains poor (Darefsky et al., 2012; Johnson et al., 2012; Koshy et al., 2012). An improved understanding of the pathogenesis and identification of the key molecular biomarkers may help in improving diagnostic accuracy and finding novel therapeutic targets for glioma to achieve better clinical outcomes.

A large body of evidence has shown the importance of tumor microenvironment (TME) in the development of tumors (Caponnetto et al., 2020; Jung et al., 2020; Yi et al., 2020). The microenvironment of glioma is composed of tumor cells, immune cells, stromal cells, and their various secreted factors. Among them, the factors secreted by tumor or immune cells, such as growth factors, chemokines, and pro-inflammatory, and anti-inflammatory factors, constitute a microenvironmental network that interact with each other to collectively regulate the process of tumorigenesis. Therefore, TME is the basis of tumor pathogenesis and is an important therapeutic target (Wood et al., 2014; Schulz et al., 2019). Previous studies have shown that the stromal cells in the TME are conducive to the tumor growth and invasion, and have an anti-tumor immune effect (Guo and Deng, 2018; Giraldo et al., 2019). Furthermore, immune cells, such as T cells and M1 and M2 macrophages, play a key role in the anti-tumor response (Bamodu et al., 2019; Heymann et al., 2019). Several immunotherapies have been shown to be effective, including immune checkpoint inhibition and therapeutic antibody applications. Some processes to serve as immunotherapy targets have been identified, such as programmed death-ligand 1 expression (Kumar et al., 2017; Koh et al., 2020), DNA mismatch repair deficiency (Overman et al., 2017; Baretti and Le, 2018), and tumor mutation burden (Chan et al., 2019; Jang et al., 2020). However, only a fraction of patients benefited from these strategies (Qi et al., 2020). Therefore, there is need to identify novel therapeutic targets to develop improved therapies with higher efficacy.

Therefore, how to accurately evaluate the dynamic composition of immune and stromal cells in TME of glioma is an important issue to address. With the development of genomic analysis, we can calculate the fraction of immune cells and stromal cells in each cancer sample and analyze the relationship between the two. We performed the ESTIMATE and CIBERSORT algorithms aiming to compute the proportion of tumor-infiltrating immune cells (TIC) and the ratio of immune and stromal components of glioma. In addition, we screened differentially expressed genes (DEGs) to identify novel prognostic indicators for immune-targeted therapy for glioma. We found annexin A1 (*ANXA1*) by evaluating the immune microenvironment of the glioma samples from The Cancer Genome Atlas (TCGA), and further verified the finding using Chinese Glioma Genome Atlas (CGGA) databases and *in vitro* experiments.

## Materials and Methods

### Antibodies and Reagents

Antibodies against ANXA1 (ab214486), B-cell lymphoma 2 (BCL-2, ab32124), BCL-2 associated X protein (BAX, ab32503), and glyceraldehyde 3-phosphate dehydrogenase (GAPDH, ab181602) were purchased from Abcam Corporation (Cambridge, MA, United States). Horseradish peroxidase-conjugated donkey anti-rabbit IgG was purchased from Bioworld (Louis Park, MN, United States). The Annexin V-FITC Apoptosis Detection Kit were purchased from Beyotime Biotechnology Technology Company (Shanghai, China). Lipofectamine 3,000 reagent was purchased from Invitrogen Corporation (Carlsbad, CA, United States). siRNA ANXA1 (siANXA1) was purchased from GenePharma Technology Company (Shanghai, China), dissolved in distilled water, and stored at −20°C. siANXA1 sequences was as follows: Forward: 5′ CCUUACCACCAGAAGCUA UTT3′, Reverse: 5′AUAGCUUCUGGUGGUAAGGTT3′.

### Cell Culture and siRNA Transfection

Human glioma cell lines, U87 MG and U251 were obtained from the Shanghai Institute of Biosciences and Cell Resources Center (Chinese Academy of Sciences, Shanghai, China). U87 MG and U251 cells were cultured in dulbecco’s modified eagle medium (DMEM) with 10% fetal bovine serum (FBS) in 6-well plates. All cells were incubated in a humidified cell incubator with 5% CO_2_ at 37°C. The cells were cultured until 70–90% confluence before transfection. Transfection was performed using Lipofectamine 3,000 reagent as per the manufacturer’s protocol. The cells were divided into siRNA group, negative control (NC) group and normal group. The siRNA group was transfected with ANXA1 siRNA and the NC group was transfected with NC siRNA for 24 h, the normal group did not receive any intervention.

### Western Blot Analysis

Cancer cell lines were seeded in 6-well plates at a density of 5 × 10^4^ cells per well and incubated over-night. Then siRNA group was transfected with ANXA1 siRNA and the NC group was transfected with NC siRNA for 24 h, the normal group did not receive any intervention. After replaced with fresh medium, the cells were continued to be incubated for 24 h. The cells were washed with phosphate-buffered saline (PBS) three times and harvested protein using RIPA lysis buffer with 1% PMSF. The protein concentration was determined using the BCA protein assay. The proteins were isolated using sodium dodecyl sulfate-polyacrylamide gel electrophoresis and transferred to a polyvinylidene fluoride membrane. After blocking it with 5% skimmed milk for 2 h and then the membrane was incubated with primary antibody (1:1,000) overnight. On the next day, the secondary antibody (1:5,000) was incubated for 2 h and the target proteins were visualized using enhanced chemiluminescence. Each experiment was done in triplicate and repeated three times independently.

### Flow Cytometry

Cancer cell lines were seeded in 6-well plates at a density of 5 × 10^4^ cells per well and incubated over-night. Then the siRNA group was transfected with ANXA1 siRNA and the NC group was transfected with NC siRNA for 24 h, the normal group did not receive any intervention. After replaced with fresh medium, the cells were continued to be incubated for 24 h. After washing the cells with PBS three times, they were digested using trypsin and collected in a 15 ml centrifuge tube. The resuspended cells were centrifuged at 1,000 *g* for 5 min. The supernatant was discarded, and cells were lightly resuspended in 195 μl Annexin V-FITC binding solution. Five microliters of annexin V-FITC was added and mixed gently. After incubating at room temperature in the dark for 20 min, flow cytometry analysis was performed to detect apoptosis on a FACSCalibur (BD Biosciences; Baltimore, MD, United States). Data were analyzed through the Flowjo software. Each experiment was done in triplicate for three independent experiments.

### Wound Healing Assay, Invasion Assay, and Colony Formation Assay

Cell migration was detected and evaluated by wounding healing assays. Briefly, Cancer cell lines were seeded in 6-well plates at a density of 5 × 10^4^ cells per well and incubated over-night. Then the siRNA group was transfected with ANXA1 siRNA and the NC group was transfected with NC siRNA for 24 h, the normal group did not receive any intervention. After replaced with fresh medium, the cells were continued to be incubated for 24 h. Scrapes were made with 200 μl sterile tips when the 6-well plates were 100% confluent. Finally, the scrape width was observed after 12 and 24 h under a microscope in different group. Invasion assay was performed to assess the ability of cell invasion. Cell treatment is as described above. Cancer cell lines were digested by trypsin and suspended again after transfected with ANXA1 siRNA and NC siRNA. Then the cells (3 × 10^4^ cells/well) were cultured in DMEM medium without 10% FBS overnight in the upper chamber in 8-μm chambers of 24-well plates containing solidified matrigel. About 800 μl of DMEM medium with 10% FBS was added to the lower chambers. The chamber was washed with PBS to remove the matrigel after 24 h. Finally, the cells were fixed with methanol for 15 min, washed with PBS three times, and the number of invasion cells were manually counted under a microscope after 0.1% crystal violet staining. Colony formation assay was performed to assess the proliferative capacity of the cells. Cell treatment is as described above. Cancer cell lines were digested by trypsin and suspended again after transfected with ANXA1 siRNA and NC siRNA. Then the cells (200 cells/well) were cultured in 6-well plates for 14 days. Finally, the cells were fixed using methanol for 15 min, washed three times with PBS, and counted using a microscope after 0.1% crystal violet staining. Colonies with more than 50 cells were considered for manual calculation. Each experiment was done in triplicate for three independent experiments.

### Bioinformatics Analysis of TCGA and CGGA Database

We downloaded the mRNA expression profiles and clinical information of low-grade glioma (LGG) and glioblastoma (GBM) samples available on TCGA^1^ dataset. mRNA expression profiles were downloaded as Fragments Per Kilobase of transcript per Million mapped reads (FPKM). Molecular subtype and treatment details were downloaded from previous study (Ceccarelli et al., 2016). Then, the “sva” package was utilized for the normalization of RNA expression profiles and to remove the batch effects between TCGA- LGG samples and TCGA-GBM samples (Leek et al., 2012). Furthermore, we downloaded 749 glioma samples and clinical information from the CGGA,^2^ which were used as a validation dataset. mRNA expression profiles were downloaded as Expectation Maximization (RSEM). The “sva” package was performed to remove the batch effects between different glioma samples.

### Calculation of Immune and Stromal

The immune and stromal scores of each glioma sample from TCGA were calculated using “limma” and “estimate” packages in R language 3.6.1, to evaluate the proportion of immune and stromal components in the TME. The higher the score, the larger the proportion of the corresponding component in the TME. According to the median score achieved, the samples were divided into high and low score groups.

### Identification of DEGs and Survival Analysis

Differential expression analysis was performed to explore the differences in gene expression between the high and low score groups. Differentially expressed genes (DEGs) were analyzed using “limma” package in R and screened by the comparison between the high-score group and the low-score group. DEGs were visualized using “pheatmap” package in R. DEGs with |log_2_ fold change| > 2 and false discovery rate (FDR) < 0.01 were considered significant. Survival analysis was performed using “survival” and “survminer” packages, to verify the difference in survival rate between the high and low score groups. The survival curve was plotted using the Kaplan–Meier method, and log rank test was used assess the statistical significance. A *p-*value < 0.05 was considered statistically significant.

### Protein-Protein Interaction Network Construction and Cox Regression Analysis

Protein-protein interaction (PPI) network of DEGs was constructed using the STRING database^3^ and Cytoscape software (Otasek et al., 2019). Nodes with confidence of interactive relationship greater than 0.95 were screened for network construction. Univariate Cox regression analysis was performed using the “survival” package to screen the genes that were closely associated with the prognosis.

### Differential Expression Analysis Between TIC and ANXA1 Expression

The TIC profile of gliomas was estimated using the CIBERSORT computational method available in R. Subsequently, the tumor samples were screened for quality and only those with *p* < 0.05 were selected for subsequent analysis. The visualization of TIC profile was constructed using the “vioplot” and “barplot” package. The differential expression analysis between ANXA1 expression and immune cells was performed using “limma” package of R language.

### Clinical Outcomes and Clinicopathological Features With ANXA1 Expression

Survival analysis of clinical outcomes was performed using “survival” and “survminer” packages, to verify the difference in survival rate between the high and low ANXA1 expression groups. The survival curve was plotted using the Kaplan–Meier method, and log rank test was used assess the statistical significance. A *p-*value < 0.05 was considered statistically significant. The relationship of clinicopathological features and ANXA1 expression were visualized using “pheatmap” package in R.

### Mutation and Prognostic Analysis of ANXA1 Expression

The mutation analysis of ANXA1 was performed using cBioPortal.^4^ Differential expression analysis was performed using Gene Expression Profiling Interactive Analysis (GEPIA)^5^ database. The overall survival and disease-free survival were also analyzed using GEPIA. The protein expression level of ANXA1 analysis was performed using Human Protein Atlas (HPA).^6^

### Statistical Analysis

Statistical analysis and charts preparation were carried out using R (version 3.6.3) and SPSS (version 25). Student’s *t*-test was used for comparison between the two groups, and variance analysis was used for data comparison between multiple groups. *p*-values < 0.05 were considered statistically significant.

## Results

### Data Filtering and Processing

**FIGURE 1:**
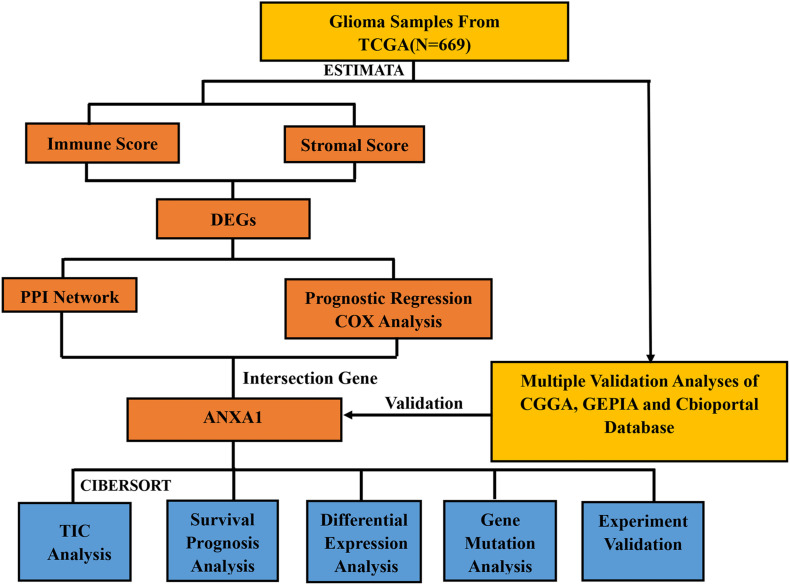
Flowchart illustrating the study design.

The study design is showed in Figure 1. We downloaded mRNA expression profiles and clinical information of 529 LGG samples and 169 GBM samples from TCGA, out of which 553 cases were recorded with detailed clinical information. Likewise, normalized mRNA expression profiles and clinical information for 458 LGG samples and 291 GBM samples were collected from the CGGA. The relevant clinical information of the patients is shown in Table 1.

**TABLE 1:** Demographic data and clinical factors.

Parameters	TCGA (*N* = 553)	CGGA (*N* = 749)
**Age**		
≤40	266 (48.10%)	342 (45.66%)
>40	287(51.90%)	407 (54.34%)
**Gender**		
Male	232 (41.95%)	442 (59.01%)
Female	321 (58.05%)	307 (40.99%)
**Tumor grade**		
WHO II	197 (35.62%)	218 (29.11%)
WHO III	217 (39.24%)	240 (32.04%)
WHO IV	139 (25.14%)	291 (38.85%)
**Grade malignance**		
Low grade glioma	414 (74.86%)	458 (61.15%)
Glioma	139 (25.14%)	291 (38.85%)
**IDH mutation**		
Mutation	345 (62.39%)	410 (54.74%)
Wild	208 (37.61%)	339 (45.26%)
**1p/19q codeletion**		
No codeletion	412 (74.50%)	594 (79.31%)
codeletion	141 (25.50%)	155 (20.69%)
**Chemotherapy**		
No	185 (33.45%)	229 (30.57%)
Yes	368 (66.55%)	520 (69.43%)
**Radiotherapy**		
No	164 (29.66%)	124 (16.56%)
Yes	389 (70.34%)	625 (83.44%)

### Immune and Stromal Scores Are Associated With Prognosis of Glioma Patients

**FIGURE 2:**
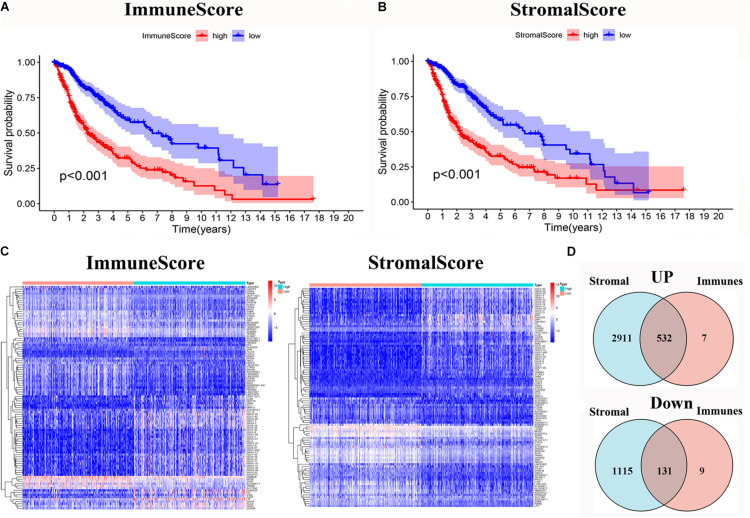
Identification of DEGs based on immune score and stromal score. **(A)** Survival analysis of glioma between high and low Immune Score groups (*p* < 0.001). **(B)** Survival analysis of glioma between high and low Stromal Score groups (*p* < 0.001). **(C)** Heatmap of the 100 DEGs with the most significant *p-*values compared between the between the high and low immune score groups; Heatmap of the 100 DEGs with the most significant *p*-values compared between the high and low stromal score groups. **(D)** Venn diagram of the common up- and down-regulated DEG shared by Immune and Stromal Scores.

To evaluate the relationship between the Immune scores and Stromal scores and survival prognosis, survival analysis was performed for the Immune and Stromal scores. The ESTIMATE algorithms were used to calculate the proportion of the number of immune and stromal components in glioma samples from TCGA. According to the median score of each sample, the patients were divided into high and low score groups. The Immune (Figure 2A) and Stromal (Figure 2B) scores were found to be negatively associated with the overall survival rate (*p* < 0.001). This suggests that the immune and stromal components of the TME can be used to predict the survival of glioma patients.

### Identification of DEGs Depended on the Immune and Stromal Scores

The DEGs were determined by differential expression analysis of immune cells and stromal cells in the TME compared between high- and low-score samples and the 100 DEGs with the most significant *p*-values was visualized using heatmap analysis (Figure 2C). A total of 679 and 4,689 DEG were screened from the Immune score group and Stromal score group, respectively. The Venn diagram analysis showed that 532 upregulated genes and 131 downregulated genes overlapped in the Immune score and Stromal score groups (Figure 2D).

### Intersectional Analysis of PPI Network and Univariate Cox Regression

**FIGURE 3:**
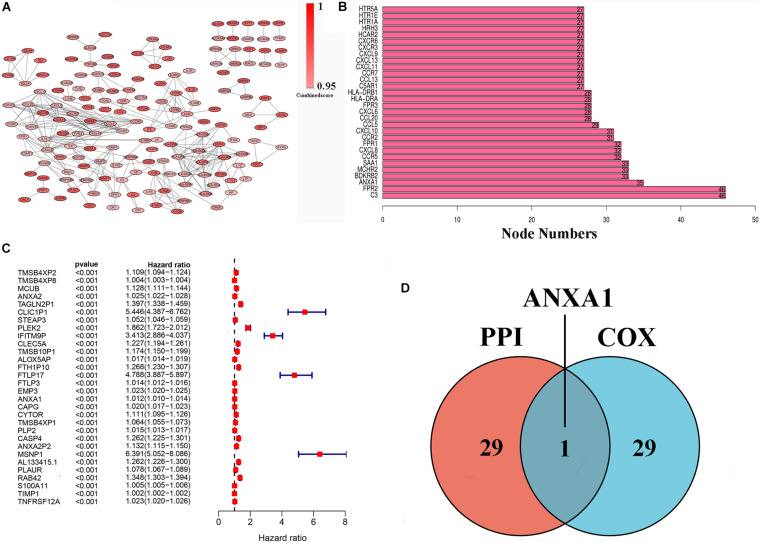
PPI network and univariate Cox regression analysis of DEG. **(A)** PPI network was constructed by nodes with interaction confidence value > 0.95. **(B)** The top 30 genes in the order based on the number of adjacent nodes. **(C)** Univariate Cox regression analysis of DEGs, listing the top 30 significant factors with *p* < 0.0001. **(D)** Venn plot showing the common gene, ANXA1 shared by PPI and univariate Cox regression analysis.

To further indicate its potential mechanism, we used Cytoscape software to construct a PPI network based on the STRING database. The PPI network of DEGs is shown in Figure 3A, and the histogram of the first 30 genes sort by the node numbers is displayed in Figure 3B. Univariate Cox regression analysis is performed to identify the top 30 significant genes ranked by the *p*-value among of the 633 DEG in glioma samples (Figure 3C). A Venn diagram between the first 30 leading nodes of the PPI network and the top 30 genes obtained from the univariate Cox regression analysis, were displayed, and only ANXA1 was found in the overlapped region from the above analyses (Figure 3D). These results showed that the ANXA1 cloud be a prognostic indicator of glioma.

### ANXA1 Is Associated With the Proportion of TIC

**FIGURE 4:**
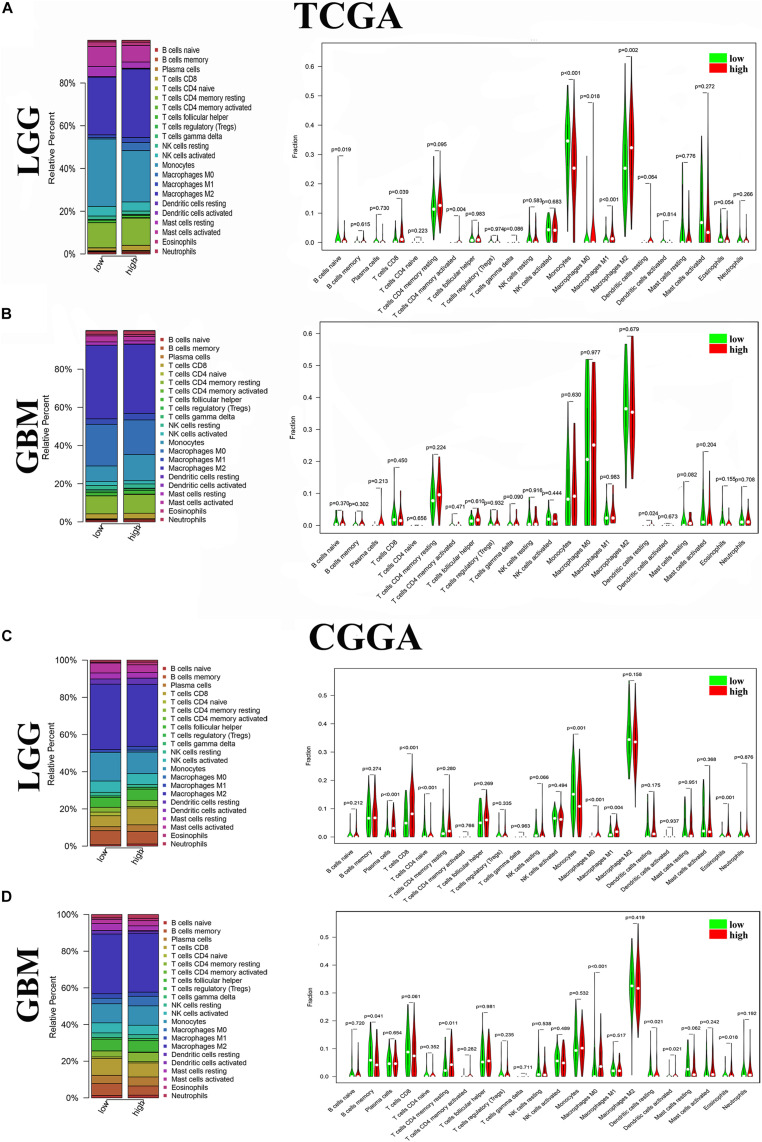
Distribution of immune cells between high and low ANXA1 expression groups. **(A)** Proportions of immune cells and violin plot of LGG glioma samples from TCGA database. **(B)** Proportions of immune cells and violin plot of GBM glioma samples from TCGA database. **(C)** Proportions of immune cells and violin plot of LGG glioma samples from CGGA database. **(D)** Proportions of immune cells and violin plot of GBM glioma samples from CGGA databases.

To further verify the association of ANXA1 expression with the TME, the proportion of TIC was analyzed using CIBERSORT computing methods, and 22 types of immune-cell distribution levels in glioma samples were built (Figure 4). Immune cells of T cells CD8, Monocytes, Macrophages M0 and Macrophages M1 have significant differences between different ANXA1 expression groups in TCGA LGG samples and CGGA LGG samples (Figures 4A,C), while immune cells of dendritic cells resting has significant differences in TCGA GBM samples and CGGA GBM samples (Figures 4B,D). The kinds of TICs correlated with ANXA1 expression in TCGA and CGGA database were described in the Table 2. These results further indicated that ANXA1 was involved in the immune activity of TME and the maintenance of cell-mediated immunity in the LGG.

**TABLE 2:** Kinds of TICs correlated with ANXA1expression in TCGA and CGGA database.

Cells of TICs	*P*-value	Project
B cells naive	0.018895	TCGA-LGG
T cells CD8	0.039071	TCGA-LGG
T cells CD4 memory activated	0.003775	TCGA-LGG
Monocytes	1.58E-05	TCGA-LGG
Macrophages M0	0.0176	TCGA-LGG
Macrophages M1	2.17E-05	TCGA-LGG
Macrophages M2	0.00153	TCGA-LGG
Dendritic cells resting	0.023647	TCGA-GBM
Plasma cells	5.57E-05	CGGA-LGG
T cells CD8	0.000473	CGGA-LGG
T cells CD4 naive	0.000521	CGGA-LGG
Monocytes	0.000779	CGGA-LGG
Macrophages M0	0.000119	CGGA-LGG
Macrophages M1	0.003922	CGGA-LGG
Eosinophils	0.001359	CGGA-LGG
B cells memory	0.040896	CGGA-GBM
T cells CD4 memory resting	0.010803	CGGA-GBM
Macrophages M0	0.000285	CGGA-GBM
Dendritic cells resting	0.021202	CGGA-GBM
Dendritic cells activated	0.021213	CGGA-GBM
Eosinophils	0.017902	CGGA-GBM

*LGG, low grade glioma; GBM, glioblastoma.*

### Different ANXA1 Expression Profiles Had Distinct Clinical Outcomes and Clinicopathological Features

**FIGURE 5:**
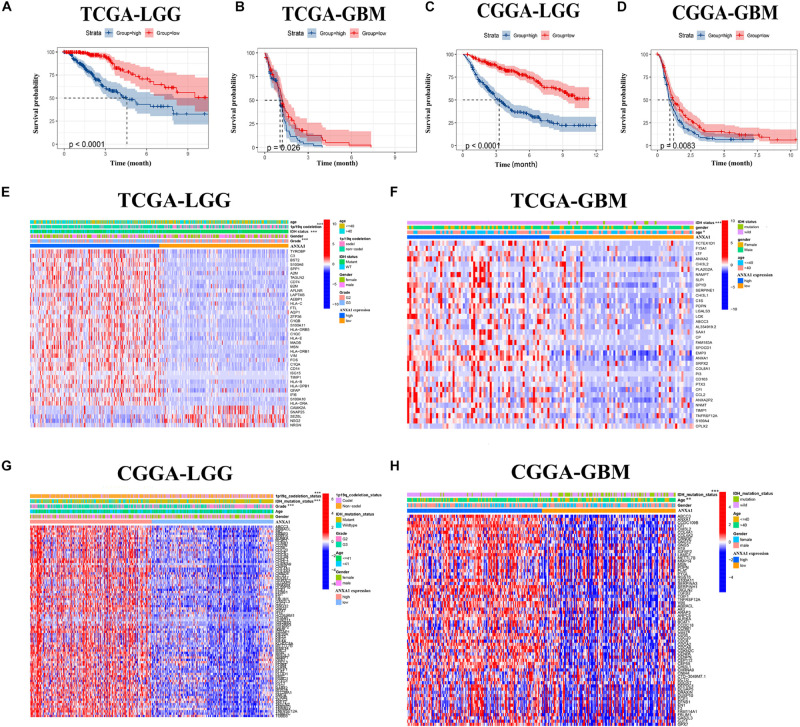
The prognostic analysis and correlation of ANXA1 expression with clinical characteristics in glioma patients through the TCGA and CGGA database. **(A,B)** The prognostic analysis of ANXA1 in the LGG and GBM glioma samples from TCGA database. **(C,D)** The prognostic analysis of ANXA1 in the LGG and GBM glioma samples from CGGA database. **(E,F)** Different ANXA1 Expression Profiles and Clinicopathological Features in the LGG and GBM samples from the TCGA database; **(G,H)** Different ANXA1 Expression Profiles and Clinicopathological Features in the LGG and GBM samples from the CGGA database; *p < 0.05; **p < 0.01; ***p < 0.001.

To verify the relationship between ANXA1 expression and survival prognosis, all glioma samples were divided into high ANXA1 expression and low ANXA1 expression groups based on the median ANXA1 expression. Glioma patients with higher ANXA1 expression, including LGG and GBM samples, had a significantly shorter survival rate than those with a lower expression from the TCGA database (*P* < 0.05) (Figures 5A,B); The survival analysis results of CGGA were consistent with those of TCGA (*P* < 0.01) (Figures 5C,D). Further we compared the clinicopathological features between the high and low ANXA1 expression group. The high ANXA1 expression group was significantly correlated with higher grade (*P* < 0.001), IDH wild status (*P* < 0.001) and 1p19q codeletion (*P* < 0.001) in LGG samples (Figure 5E); In the GBM samples, the high ANXA1 expression group is significantly correlated with older (*P* < 0.05) and IDH wild status (*P* < 0.001) from TCGA database (Figure 5F). In the CGGA database, the high ANXA1 expression group also was significantly correlated with IDH wild status (*P* < 0.001) and 1p19q codeletion (*P* < 0.001) in LGG samples (Figure 5G); Meanwhile, it also was significantly correlated with older (*P* < 0.01) and IDH wild status (*P* < 0.001) in GBM samples (Figure 5H). These findings clearly indicate that ANXA1 expression in TME is negatively associated with the prognosis of glioma. In particular, the expression of ANXA1 was found to increase with the progression of tumor grade and malignancy.

### Mutation and Prognostic Analysis of ANXA1 Expression in Multiple Databases

**FIGURE 6:**
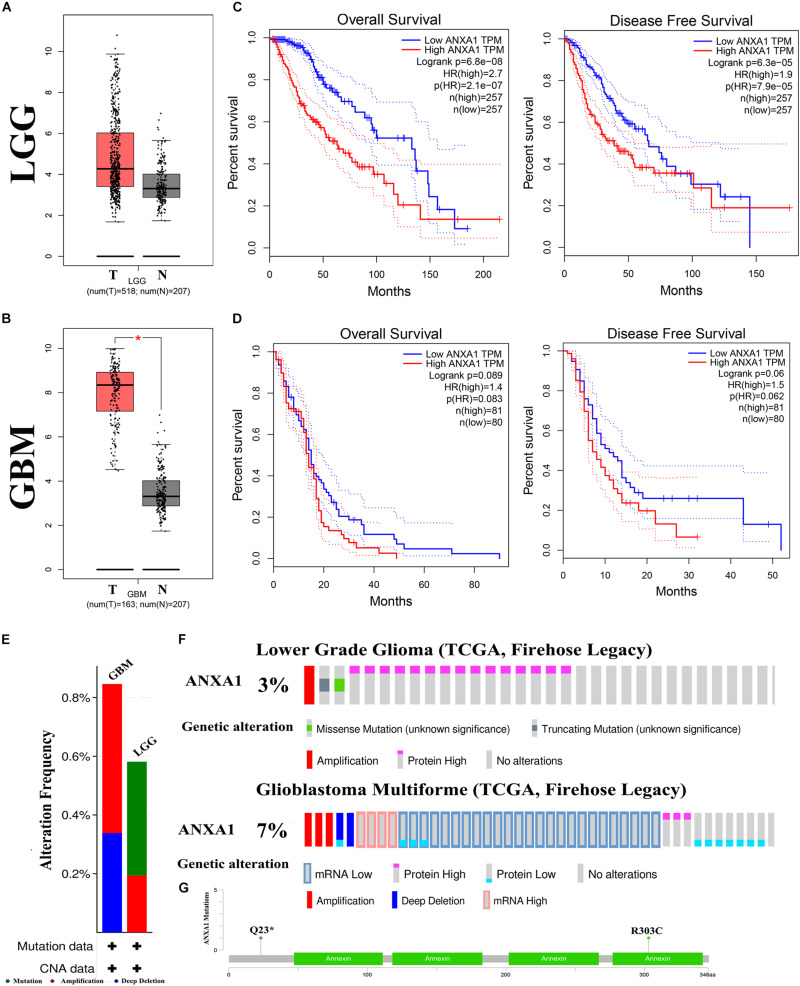
Mutation and prognostic analysis of ANXA1 Expression in GEPIA and cBioPortal databases. **(A,B)** According the GEPIA database, the result of differential expression analysis of ANXA1 expression. Red represents cancer tissues and gray represents normal tissues in the LGG and GBM samples (*p < 0.05). **(C,D)** The overall survival and disease-free survival rate of ANXA1. **(E)** The mutation type of ANXA1. **(F)** The mutation analysis of ANXA1. **(G)** The common mutation site are Q23 and R303C.

To further investigate the role of ANXA1 expression in glioma, we performed differential expression and mutation analyses of ANXA1 using GEPIA (gepia.cancer-pku.cn) and cBioPortal (see text footnote 4) databases. According to the GEPIA database, the results of differential expression analysis revealed an increased expression of ANXA1 in the tumor samples compared with normal samples in LGG samples (Figure 6A) and GBM samples (Figure 6B). The overall survival and disease-free survival rate of patients with low ANXA1 expression were significantly better than those with high expression in LGG samples (*P* < 0.001) (Figure 6C) and GBM samples (Figure 6D). The purpose of mutation analysis is to better identify the mutation status and sites of ANXA1 in glioma, and mutation sites are common targets of gene therapy. The mutation type of ANXA1 in GBM was mainly amplification, while in low-grade gliomas was mainly mutation (Figure 6E). The mutation analysis of ANXA1 showed that the mutation frequency of ANXA1 were 3 and 7% in LGG and GBM samples, respectively (Figure 6F). Mutation frequency of ANXA1 was higher in GBM samples than in the LGG samples and the common mutation sites were Q23 and R303C (Figure 6G). These results further validated that ANXA1 was closely related to prognosis and could serve as a therapeutic target in glioma.

### Correlation of ANXA1 Expression With Immune Checkpoint Markers of Glioma

**FIGURE 7:**
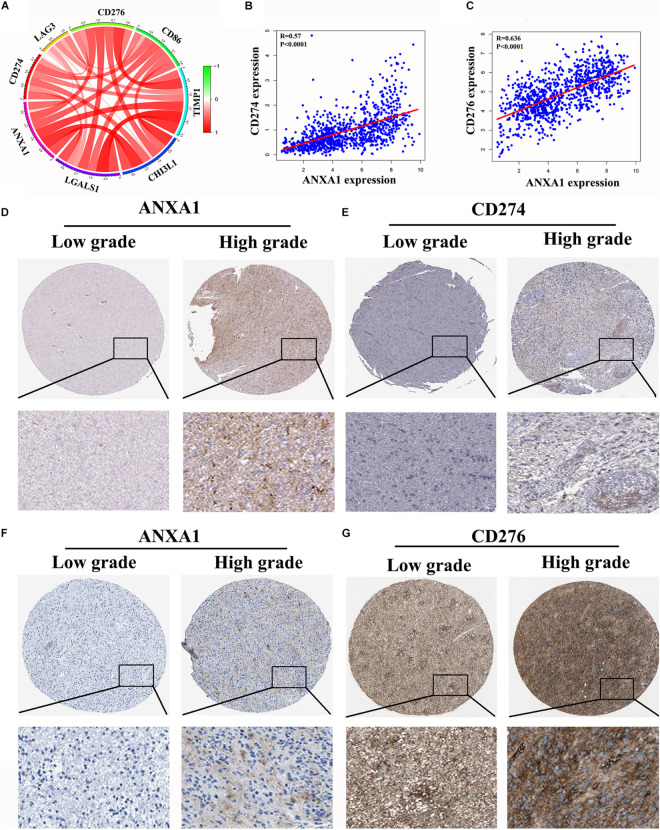
Correlation of ANXA1 Expression with Immune Checkpoint Markers of Glioma. **(A)** In the CGGA dataset, ANXA1 was positively correlated with Immune Checkpoint Markers. **(B,C)** ANXA1 was strongly related with CD274 and CD276 expression. **(D,E)** ANXA1 and CD274 expression were positive in high grade gliomas, and there is a certain correlation between their expressions. **(F,G)** ANXA1 and CD276 protein also were simultaneously overexpressed in high grade gliomas.

In the CGGA dataset, we analyzed relation between the ANXA1 expression and immune checkpoint markers (CD274, CD276, LAG3, CD86, TIMP1, and CHI3L1). We found that the ANXA1 expression was positively correlated with these markers (Figure 7A) and strongly related with CD274 and CD276 expression (Figures 7B,C). Then we download the low-grade and high-grade glioma samples of the same patients from HPA database. The result showed that ANXA1 and CD274 expression (patient id:122; 3,226) were positive in high grade gliomas, and there is a certain correlation between their expressions (Figures 7D,E). ANXA1 and CD276 (Patient id: 3,174; 3,241) also were overexpressed in high grade gliomas (Figures 7F,G). These results indicated ANXA1 influenced the tumor microenvironment by regulating these tumor immune genes.

### Silencing ANXA1 Expression Promotes Cell Apoptosis by Targeting BCL-2 and Bax

**FIGURE 8:**
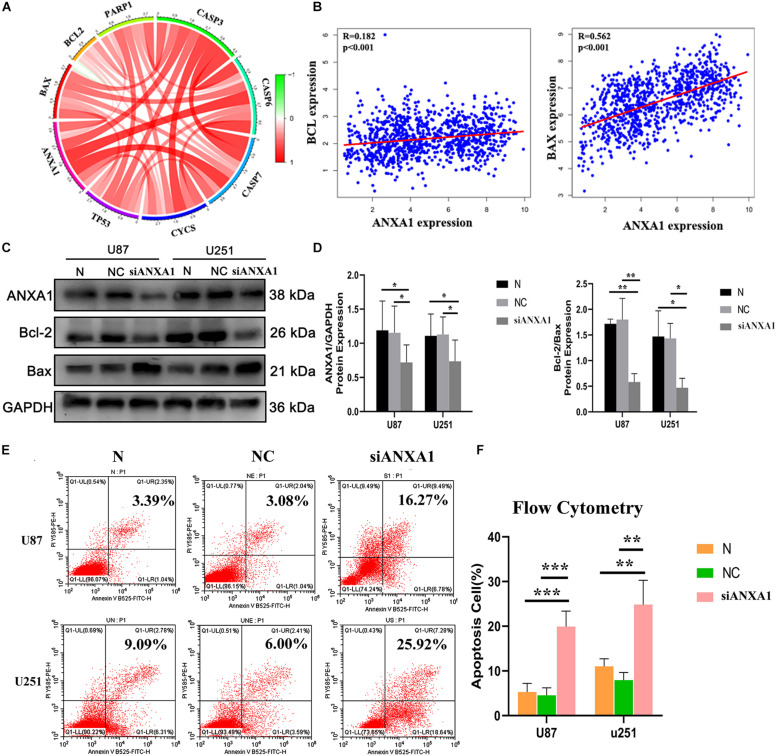
Silencing ANXA1 expression promotes cell apoptosis by targeting Bcl-2 and Bax. In the CGGA dataset, **(A)** ANXA1 expression was positively correlated with apoptosis markers and strongly related with BAX expression **(B)**. **(C,D)** The analysis showed that silencing ANXA1 expression downregulated bcl-2 and upregulated Bax protein levels (*p* < 0.05). **(E,F)** The flow cytometry analysis also revealed the apoptosis rate significantly increased in the siANXA1 group (*P* < 0.01). *p < 0.05; **p < 0.01; ***p < 0.001.

The TME can influence the cell proliferation and death of tumor cells, and apoptosis is a common form of cell death (Su et al., 2015; Yaacoub et al., 2016). Therefore, we further studied the relationship between ANXA1 and cell apoptosis. In the CGGA dataset, we analyzed relation between the ANXA1 expression and apoptosis markers (BAX, BCL-2, CASP3, CYCS, TP53, and PAPR). We found that the ANXA1 expression was positively correlated with these markers (Figure 8A) and strongly related with BAX expression (Figure 8B). To investigate the apoptotic pathways involved in cell death due to inhibition of ANXA1 expression, we performed western blot to analyze the change in expression levels of the protein involved in cell apoptosis caused by silencing ANXA1 expression in U87 and U251 cell lines. The results showed that in cells transfected with siANXA1, bcl-2 expression was downregulated and Bax was upregulated, which regulated the apoptosis (*P* < 0.05) (Figures 8C,D). Then the flow cytometry assays were performed to assess the cell apoptosis. the flow cytometry analysis also revealed a significantly higher rate of apoptosis in the siANXA1 group (*P* < 0.01) (Figures 8E,F). These results verified the hypothesis that inhibition of ANXA1 expression induced cell apoptosis in U87 and U251 cells.

### Silencing ANXA1 Reduced Migration, Invasion, and Proliferation in Glioma Cell Lines

**FIGURE 9:**
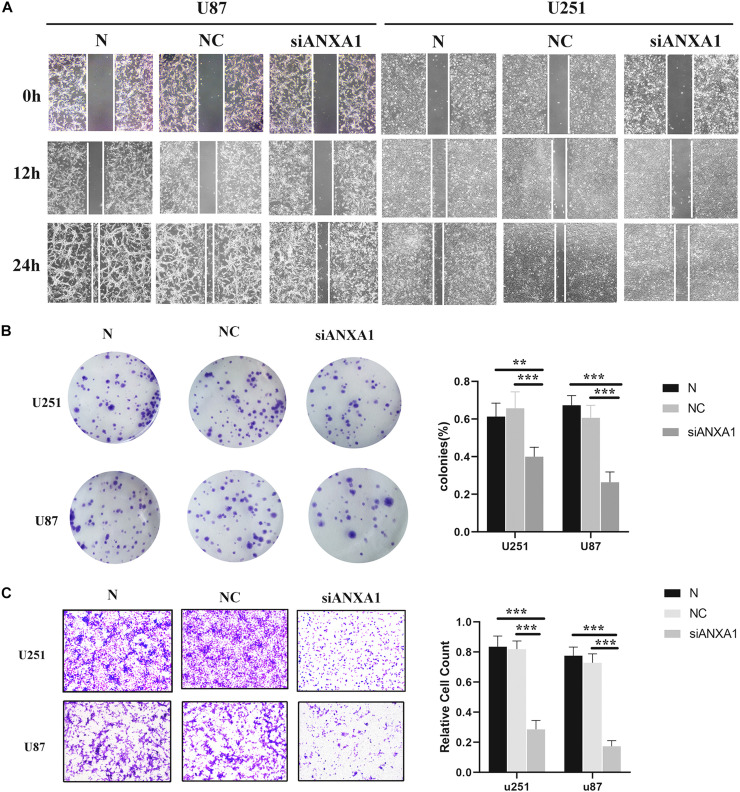
Wound healing assay, Transwell assay, and colony formation assay. **(A)** The migration ability of siANXA1 group was significantly reduced compared with NC and normal group (100X). **(B)** The cell cloning ability of siANXA1 group was also significantly reduced compared with NC and normal group. **(C)** The invasion ability of siANXA1 group cells was reduced compared with that of NC and normal group (100X). **p < 0.01; ***p < 0.001.

Cell migration and invasion are important steps in tumor growth and therefore we evaluated the effects of siANXA1 on migration and invasion in U87 and 251 cell lines. Wound healing assay showed that the migration ability of the siANXA1 group was significantly reduced compared to that of the normal and NC group (Figure 9A). Furthermore, the colony formation assay indicated that ability to form colonies was significantly reduced in the siANXA1 group compared to that in the normal and NC group (Figure 9B). Finally, the Transwell assay showed that the siANXA1 group was less invasive than normal and NC group (*P* < 0.05) (Figure 9C). These experimental results suggested that the inhibition of ANXA1 expression could reduce the metastatic potential of gliomas, which further verified that ANXA1 expression was related to malignancy in gliomas.

## Discussion

Despite the combination of surgery, radiotherapy, and chemotherapy, patients with glioma show poor clinical prognosis. Therefore, we need to find more effective treatment methods to improve survival in these patients. Many studies have shown that the TME plays an important role in the growth and invasion of glioma (Chang et al., 2016; Qian et al., 2018; Meng et al., 2019). Previous studies have shown that therapies targeting the tumor microenvironment have achieved good results, such as anti-programmed death-ligand 1 therapy (Ruan et al., 2019; Ene et al., 2020) and T cell immunotherapy (Brown et al., 2016; Choi et al., 2019). Moreover, immunotherapy has been shown to be effective in other cancers such as lung carcinoma and melanoma (Topalian et al., 2012). Upon evaluating the glioma samples in the TCGA database, our results showed that the immune components in TME contributed to the prognosis of glioma. Additionally, we found that besides the proportion of immune and stromal components of TME, tumor grade and histopathologic characteristics were significantly related to the malignancy of glioma. These results highlight the importance of exploring the interaction between tumor cells and immune cells and researching new therapeutic targets for the development of more effective treatments.

The discovery of lymphatic system in the central nervous system has provided a new theoretical basis and opened up novel prospects for immunotherapy for brain tumors (Louveau et al., 2015). Multiple immune-related signaling pathways, such as the phosphatidyl inositol-3-kinase/Akt pathway, IGF pathway, and programmed death-ligand 1 signaling, individually or collectively impact the TME of glioma (Kravchenko et al., 2015; Wang et al., 2016; Cai et al., 2018). Based on transcriptome sequencing analysis of the glioma samples from TCGA database, we found that increased ANXA1 expression was significantly associated with advanced clinicopathological features and poor prognosis in patients with glioma. These results indicated that ANXA1 could be a prognostic indicator and an immunotherapy marker for TME in glioma.

ANXA1 is a membrane-localized protein that binds phospholipids and has an anti-inflammatory activity. It plays important roles in the innate immune response as an effector of glucocorticoid-mediated responses and a regulator of the inflammatory processes (Arcone et al., 1993; Leoni et al., 2015). It contributes to the adaptive immune response by enhancing the signaling cascades that are triggered by T-cell activation, regulates differentiation and proliferation of activated T-cells, promotes the differentiation of T-cells into Th1 cells, and negatively regulates their differentiation into Th2 cells (D’Acquisto et al., 2007). ANXA1 has been shown to be abnormally expressed in the immune microenvironment of cancer (Moraes et al., 2017, 2018), and antagonizing ANXA1 expression has often achieved good results (Hatakeyama et al., 2011; Gastardelo et al., 2014). Therefore, we further analyzed the relationship between ANXA1 expression and TME. The CIBERSORT computing method for the evaluating the proportion of TIC revealed a positive correlation between T-cell activation and ANXA1 expression in LGG glioma samples, suggesting that ANXA1 could be involved in the maintenance of cell-mediated immunity in the TME.

ANXA1 is differentially expressed in a variety of tumors and a number of genome-sequencing studies have shown that ANXA1 is highly expressed in gliomas (Ruano et al., 2008; Naryzhnyi et al., 2014; Mallawaaratchy et al., 2015; Qiu et al., 2020), but intensive research on ANXA1 function in glioma was lacking. To explore the underlying mechanism of the ANXA1 in TME, we investigated the relationship between ANXA1 and several important immune checkpoint markers through the HPA database. The result showed that NXA1 expression was positively correlated with CD274 and CD276 expression, which indicated ANXA1 could influenced the tumor microenvironment by regulating these tumor immune genes. Then we investigated the role of ANXA1 in cell proliferation and growth through *in vitro* experiments. The *in vitro* experiments showed that suppressing ANXA1 expression could significantly inhibit the proliferation and motility of glioma cells and promote cell death via apoptosis. Overall, these findings have proved that ANXA1, a key gene in the TME, can be an effective target for treatment of glioma.

These results suggest that targeted tumor microenvironment therapy for glioma can achieve satisfactory results. ANXA1 inhibition needs to be explored further *in vitro* and *in vivo* to better understand the efficacy of targeting this protein in suppressing growth and tumor progression of glioma. In future, continued screening of core genes in the glioma immune microenvironment can help us develop novel targets more accurately and provide a theoretical basis for the pathological mechanism of glioma.

## Data Availability Statement

The datasets presented in this study can be found in online repositories. The names of the repository/repositories and accession number(s) can be found in the article/Supplementary material.

## Author Contributions

ZL, EY, XL, and HW designed the study and wrote the initial draft of the manuscript. MW, JC, CY, HZ, JR, KW, YZ, and LH contributed to data analysis. QZ and SY reviewed and edited the manuscript. All authors read and approved the manuscript.

## Conflict of Interest

The authors declare that the research was conducted in the absence of any commercial or financial relationships that could be construed as a potential conflict of interest.

## Publisher’s Note

All claims expressed in this article are solely those of the authors and do not necessarily represent those of their affiliated organizations, or those of the publisher, the editors and the reviewers. Any product that may be evaluated in this article, or claim that may be made by its manufacturer, is not guaranteed or endorsed by the publisher.

## Footnotes

1
https://portal.gdc.cancer.gov/

2
http://www.cgga.org.cn/

3
https://string-db.org/

4
www.cbioportal.org

5
http://gepia.cancer-pku.cn/

6
https://www.proteinatlas.org/

## Abbreviations

ANXA1: annexin A1
bcl-2: B-cell lymphoma-2
Bax: bcl-2-associated X protein
CGGA: Chinese Glioma Genome Atlas
DEGs: differentially expressed genes
FBS: fetal bovine serum
GEPIA: Gene Expression Profiling Interactive Analysis
GSEA: gene set enrichment analysis
GBM: glioblastoma
LGG: low grade glioma
NC: negative control
PBS: phosphate buffer saline
PPI: protein-protein interaction
TCGA: The Cancer Genome Atlas
TIC: tumor infiltrating cells
TME: tumor microenvironment.

## References

[B1] Arcone R. Arpaia G. Ruoppolo M. Malorni A. Pucci P. Marino G. (1993). Structural characterization of a biologically active human lipocortin 1 expressed in *Escherichia coli*. *Eur. J. Biochem.* 211 347–355. 10.1111/j.1432-1033.1993.tb19904.x 8425544

[B2] Bamodu O. A. Kuo K. T. Wang C. H. Huang W. C. Wu A. T. H. Tsai J. T. (2019). Astragalus polysaccharides (PG2) enhances the M1 polarization of macrophages, functional maturation of dendritic cells, and T cell-mediated anticancer immune responses in patients with Lung cancer. *Nutrients* 11:2264. 10.3390/nu11102264 31547048 PMC6836209

[B3] Baretti M. Le D. T. (2018). DNA mismatch repair in cancer. *Pharmacol. Ther.* 189 45–62. 29669262 10.1016/j.pharmthera.2018.04.004

[B4] Brown C. E. Alizadeh D. Starr R. Weng L. Wagner J. R. Naranjo A. (2016). Regression of glioblastoma after chimeric antigen receptor T-cell therapy. *N. Engl. J. Med.* 375 2561–2569. 28029927 10.1056/NEJMoa1610497 PMC5390684

[B5] Cai J. Chen Q. Cui Y. Dong J. Chen M. Wu P. (2018). Immune heterogeneity and clinicopathologic characterization of IGFBP2 in 2447 glioma samples. *Oncoimmunology* 7:e1426516. 10.1080/2162402x.2018.1426516 29721393 PMC5927515

[B6] Caponnetto F. Dalla E. Mangoni D. Piazza S. Radovic S. Ius T. (2020). The miRNA content of exosomes released from the glioma microenvironment can affect malignant progression. *Biomedicines* 8:564. 10.3390/biomedicines8120564 33287106 PMC7761654

[B7] Ceccarelli M. Barthel F. P. Malta T. M. Sabedot T. S. Salama S. R. Murray B. A. (2016). Molecular profiling reveals biologically discrete subsets and pathways of progression in diffuse glioma. *Cell* 164 550–563. 26824661 10.1016/j.cell.2015.12.028 PMC4754110

[B8] Chan T. A. Yarchoan M. Jaffee E. Swanton C. Quezada S. A. Stenzinger A. (2019). Development of tumor mutation burden as an immunotherapy biomarker: utility for the oncology clinic. *Ann. Oncol.* 30 44–56. 10.1093/annonc/mdy495 30395155 PMC6336005

[B9] Chang A. L. Miska J. Wainwright D. A. Dey M. Rivetta C. V. Yu D. (2016). CCL2 produced by the glioma microenvironment is essential for the recruitment of regulatory T cells and myeloid-derived suppressor cells. *Cancer Res.* 76 5671–5682. 10.1158/0008-5472.can-16-0144 27530322 PMC5050119

[B10] Choi B. D. Maus M. V. June C. H. Sampson J. H. (2019). Immunotherapy for glioblastoma: adoptive T-cell Strategies. *Clin. Cancer Res.* 25 2042–2048. 10.1158/1078-0432.ccr-18-1625 30446589 PMC6445734

[B11] D’Acquisto F. Merghani A. Lecona E. Rosignoli G. Raza K. Buckley C. D. (2007). Annexin-1 modulates T-cell activation and differentiation. *Blood* 109 1095–1102. 10.1182/blood-2006-05-022798 17008549 PMC1855438

[B12] Darefsky A. S. King J. J. Dubrow R. (2012). Adult glioblastoma multiforme survival in the temozolomide era: a population-based analysis of surveillance, epidemiology, and end results registries. *Cancer-Am. Cancer Soc.* 118 2163–2172. 10.1002/cncr.26494 21882183 PMC3235223

[B13] Ene C. I. Kreuser S. A. Jung M. Zhang H. Arora S. White Moyes K. (2020). Anti-PD-L1 antibody direct activation of macrophages contributes to a radiation-induced abscopal response in glioblastoma. *Neuro. Oncol.* 22 639–651. 10.1093/neuonc/noz226 31793634 PMC7229244

[B14] Gastardelo T. S. Cunha B. R. Raposo L. S. Maniglia J. V. Cury P. M. Lisoni F. C. (2014). Inflammation and cancer: role of annexin A1 and FPR2/ALX in proliferation and metastasis in human laryngeal squamous cell carcinoma. *PLoS One* 9:e111317. 10.1371/journal.pone.0111317 25490767 PMC4260827

[B15] Giraldo N. A. Sanchez-Salas R. Peske J. D. Vano Y. Becht E. Petitprez F. (2019). The clinical role of the TME in solid cancer. *Br. J. Cancer* 120 45–53. 10.1038/s41416-018-0327-z 30413828 PMC6325164

[B16] Guo S. Deng C. X. (2018). Effect of stromal cells in tumor microenvironment on metastasis initiation. *Int. J. Biol. Sci.* 14 2083–2093. 10.7150/ijbs.25720 30585271 PMC6299363

[B17] Hatakeyama S. Sugihara K. Shibata T. K. Nakayama J. Akama T. O. Tamura N. (2011). Targeted drug delivery to tumor vasculature by a carbohydrate mimetic peptide. *Proc. Natl. Acad. Sci. U S A.* 108 19587–19592. 10.1073/pnas.1105057108 22114188 PMC3241764

[B18] Heymann M. F. Lezot F. Heymann D. (2019). The contribution of immune infiltrates and the local microenvironment in the pathogenesis of osteosarcoma. *Cell Immunol.* 343:103711. 10.1016/j.cellimm.2017.10.011 29117898

[B19] Jang B. S. Han W. Kim I. A. (2020). Tumor mutation burden, immune checkpoint crosstalk and radiosensitivity in single-cell RNA sequencing data of breast cancer. *Radiother. Oncol.* 142 202–209. 10.1016/j.radonc.2019.11.003 31767471

[B20] Johnson D. R. Ma D. J. Buckner J. C. Hammack J. E. (2012). Conditional probability of long-term survival in glioblastoma: a population-based analysis. *Cancer-Am. Cancer Soc.* 118 5608–5613. 10.1002/cncr.27590 22569786

[B21] Jung Y. Cackowski F. C. Yumoto K. Decker A. M. Wang Y. Hotchkin M. (2020). Abscisic acid regulates dormancy of prostate cancer disseminated tumor cells in the bone marrow. *Neoplasia* 23 102–111. 10.1016/j.neo.2020.11.009 33296752 PMC7721692

[B22] Koh Y. W. Han J. H. Haam S. (2020). Expression of PD-L1, cancer stem cell and epithelial-mesenchymal transition phenotype in non-small cell lung cancer. *Pathology* 53 239–246. 10.1016/j.pathol.2020.07.009 33036771

[B23] Koshy M. Villano J. L. Dolecek T. A. Howard A. Mahmood U. Chmura S. J. (2012). Improved survival time trends for glioblastoma using the SEER 17 population-based registries. *J. Neurooncol.* 107 207–212. 10.1007/s11060-011-0738-7 21984115 PMC4077033

[B24] Kravchenko J. Corsini E. Williams M. A. Decker W. Manjili M. H. Otsuki T. (2015). Chemical compounds from anthropogenic environment and immune evasion mechanisms: potential interactions. *Carcinogenesis* 36(Suppl. 1), S111–S127. 26002081 10.1093/carcin/bgv033 PMC4565606

[B25] Kumar R. Yu F. Zhen Y. H. Li B. Wang J. Yang Y. (2017). PD-1 blockade restores impaired function of *ex vivo* expanded CD8(+) T cells and enhances apoptosis in mismatch repair deficient EpCAM(+)PD-L1(+) cancer cells. *Onco. Targets Ther.* 10 3453–3465. 10.2147/ott.s130131 28761354 PMC5516878

[B26] Leek J. T. Johnson W. E. Parker H. S. Jaffe A. E. Storey J. D. (2012). The sva package for removing batch effects and other unwanted variation in high-throughput experiments. *Bioinformatics* 28 882–883. 10.1093/bioinformatics/bts034 22257669 PMC3307112

[B27] Leoni G. Neumann P. A. Kamaly N. Quiros M. Nishio H. Jones H. R. (2015). Annexin A1-containing extracellular vesicles and polymeric nanoparticles promote epithelial wound repair. *J. Clin. Invest.* 125 1215–1227. 10.1172/jci76693 25664854 PMC4362251

[B28] Louveau A. Smirnov I. Keyes T. J. Eccles J. D. Rouhani S. J. Peske J. D. (2015). Structural and functional features of central nervous system lymphatic vessels. *Nature* 523 337–341. 10.1038/nature14432 26030524 PMC4506234

[B29] Mallawaaratchy D. M. Buckland M. E. Mcdonald K. L. Li C. C. Ly L. Sykes E. K. (2015). Membrane proteome analysis of glioblastoma cell invasion. *J. Neuropathol. Exp. Neurol.* 74 425–441. 10.1097/nen.0000000000000187 25853691

[B30] Meng X. Duan C. Pang H. Chen Q. Han B. Zha C. (2019). DNA damage repair alterations modulate M2 polarization of microglia to remodel the tumor microenvironment via the p53-mediated MDK expression in glioma. *Ebiomedicine* 41 185–199. 10.1016/j.ebiom.2019.01.067 30773478 PMC6442002

[B31] Moraes L. A. Ampomah P. B. Lim L. (2018). Annexin A1 in inflammation and breast cancer: a new axis in the tumor microenvironment. *Cell Adh. Migr.* 12 417–423. 30122097 10.1080/19336918.2018.1486143 PMC6363057

[B32] Moraes L. A. Kar S. Foo S. L. Gu T. Toh Y. Q. Ampomah P. B. (2017). Annexin-A1 enhances breast cancer growth and migration by promoting alternative macrophage polarization in the tumour microenvironment. *Sci. Rep.* 7:17925. 10.1038/s41598-017-17622-5 PMC5738423 29263330

[B33] Naryzhnyi S. N. Ronzhina N. L. Mainskova M. A. Beliakova N. V. Pantina R. A. Filatov M. V. (2014). [Development of barcode and proteome profiling of glioblastoma]. *Biomed. Khim.* 60 308–321. 10.18097/pbmc20146003308 25019393

[B34] Ostrom Q. T. Bauchet L. Davis F. G. Deltour I. Fisher J. L. Langer C. E. (2014). The epidemiology of glioma in adults: a “state of the science” review. *Neuro. Oncol.* 16 896–913. 10.1093/neuonc/nou087 24842956 PMC4057143

[B35] Otasek D. Morris J. H. Boucas J. Pico A. R. Demchak B. (2019). Cytoscape automation: empowering workflow-based network analysis. *Genome Biol.* 20:185. 10.1186/s13059-019-1758-4 PMC6717989 31477170

[B36] Overman M. J. Mcdermott R. Leach J. L. Lonardi S. Lenz H. J. Morse M. A. (2017). Nivolumab in patients with metastatic DNA mismatch repair-deficient or microsatellite instability-high colorectal cancer (CheckMate 142): an open-label, multicentre, phase 2 study. *Lancet Oncol.* 18 1182–1191. 10.1016/s1470-2045(17)30422-9 28734759 PMC6207072

[B37] Qi Y. Liu B. Sun Q. Xiong X. Chen Q. (2020). Immune checkpoint targeted therapy in glioma: status and hopes. *Front. Immunol.* 11:578877. 10.3389/fimmu.2020.578877 33329549 PMC7729019

[B38] Qian J. Wang C. Wang B. Yang J. Wang Y. Luo F. (2018). The IFN-gamma/PD-L1 axis between T cells and tumor microenvironment: hints for glioma anti-PD-1/PD-L1 therapy. *J. Neuroinflamm.* 15:290. 10.1186/s12974-018-1330-2 PMC6192101 30333036

[B39] Qiu H. Li Y. Cheng S. Li J. He C. Li J. (2020). A prognostic microenvironment-related immune signature via estimate (PROMISE Model) predicts overall survival of patients with glioma. *Front. Oncol.* 10:580263. 10.3389/fonc.2020.580263 33425732 PMC7793983

[B40] Ruan S. Xie R. Qin L. Yu M. Xiao W. Hu C. (2019). Aggregable nanoparticles-enabled chemotherapy and autophagy inhibition combined with Anti-PD-L1 antibody for improved glioma treatment. *Nano Lett.* 19 8318–8332. 10.1021/acs.nanolett.9b03968 31610656

[B41] Ruano Y. Mollejo M. Camacho F. I. Rodríguez, de Lope A. Fiaño C. (2008). Identification of survival-related genes of the phosphatidylinositol 3’-kinase signaling pathway in glioblastoma multiforme. *Cancer-Am. Cancer Soc.* 112 1575–1584. 10.1002/cncr.23338 18260157

[B42] Schulz M. Salamero-Boix A. Niesel K. Alekseeva T. Sevenich L. (2019). Microenvironmental regulation of tumor progression and therapeutic response in brain metastasis. *Front. Immunol.* 10:1713. 10.3389/fimmu.2019.01713 31396225 PMC6667643

[B43] Su Z. Yang Z. Xu Y. Chen Y. Yu Q. (2015). Apoptosis, autophagy, necroptosis, and cancer metastasis. *Mol. Cancer* 14:48. 10.1186/s12943-015-0321-5 25743109 PMC4343053

[B44] Topalian S. L. Hodi F. S. Brahmer J. R. Gettinger S. N. Smith D. C. McDermott D. F. (2012). Safety, activity, and immune correlates of anti-PD-1 antibody in cancer. *N. Engl. J. Med.* 366 2443–2454. 22658127 10.1056/NEJMoa1200690 PMC3544539

[B45] Wang Z. Zhang C. Liu X. Wang Z. Sun L. Li G. (2016). Molecular and clinical characterization of PD-L1 expression at transcriptional level via 976 samples of brain glioma. *Oncoimmunology* 5:e1196310. 10.1080/2162402x.2016.1196310 27999734 PMC5139638

[B46] Wood S. L. Pernemalm M. Crosbie P. A. Whetton A. D. (2014). The role of the tumor-microenvironment in lung cancer-metastasis and its relationship to potential therapeutic targets. *Cancer Treat. Rev.* 40 558–566. 10.1016/j.ctrv.2013.10.001 24176790

[B47] Yaacoub K. Pedeux R. Tarte K. Guillaudeux T. (2016). Role of the tumor microenvironment in regulating apoptosis and cancer progression. *Cancer Lett.* 378 150–159. 10.1016/j.canlet.2016.05.012 27224890

[B48] Yi K. Zhan Q. Wang Q. Tan Y. Fang C. Wang Y. (2020). PTRF/Cavin1 remodels phospholipid metabolism to promote tumor proliferation and suppress immune responses in glioblastoma by stabilizing cPLA2. *Neuro Oncol.* 23 387–399. 10.1093/neuonc/noaa255 33140095 PMC7992898

